# Three Novel Variants of CEP290 and CC2D2DA and a Link Between ZNF77 and SHH Signaling Pathway Are Found in Two Meckel-Gruber Syndrome Fetuses

**DOI:** 10.1007/s43032-021-00835-5

**Published:** 2022-01-03

**Authors:** Zhidan Hong, Xuanyi He, Fang Yu, Huanyu Liu, Xiaoli Zhang, Yuanzhen Zhang

**Affiliations:** 1 Reproductive Medicine Center, Zhongnan Hospital, Wuhan University, Wuhan, 430071 China; 2 Department of Pathology, Zhongnan Hospital, Wuhan University, Wuhan, 430071 China; 3 Department of Ultrasound, Zhongnan Hospital, Wuhan University, Wuhan, 430071 China

**Keywords:** CEP290, CC2D2A, ZNF77, Meckel-Gruber syndrome, Novel variant, SHH

## Abstract

Meckel-Gruber syndrome (MKS) is a rare lethal autosomal recessive inherited disorder. Missed diagnosis might happen in clinical works due to an unclear genotype–phenotype correlation. We analyzed two families visiting our center; the parents are normal; each of the family aborted a fetus at 12WG. Following ultrasonography and pathological examination, both were diagnosed as MKS. Whole exome sequencing identified a compound heterozygous of two novel variants of CEP290 and a heterozygous of a novel variant of CC2D2A. Frameshift mutations in ZNF77 were also detected. Western blot analyzing whole-brain tissue showed that the expression of ZNF77, CC2D2A, and CEP290 was enhanced. HEK293T transfected with over-expression wildtype/mutated ZNF77 plasmid showed that SHH was increased in wildtype ZNF77 cells, while SHH and CC2D2A were increased in mutated ZNF77 cells. Our research provided two novel pathogenic variants of CEP290 and CC2D2A and suggested that ZNF77 might promote the expression of CC2D2A and regulate the amount of SHH.

## Introduction

Meckel-Gruber Syndrome (MKS, OMIM *24,900), one of the most serious ciliopathies and the most common neural tube defect (NTDs) [1], is a rare lethal autosomal recessive inherited disorder. Globally, the incidence of MKS is 1/140000–1/13250 [2]. A couple of carriers has a possibility of 25% to produce a MKS fetus, and both two genders gain the same risk. Important systems and organs could be impacted in MKS, leading to a wide phenotypic spectrum, which a “classic triad” (occipital encephalocele, cystic kidney dysplasia, polydactyly) [3] might characterize in the majority of patients. Clinical prenatal diagnosis usually comes from transvaginal ultrasound from 11 to 16 weeks of gestation. However, MKS has a high heterogeneity. Clinical manifestations are different at the general, organic, and even tissue level among different cases. The advancement of assisted reproductive technology, the development of molecular diagnostic technology, and preimplantation genetic testing (PGT) can prevent the occurrence of this disease to a certain extent. However, the accumulation of resources of the nation is a necessary prerequisite for the research of this type of genetic disease.

From the perspective of genetics, variants from at least 14 germlines have been already reported to cause MKS: *MKS1*, *TMEM216*, *TMEM67*, *CEP290*, *RPGRIP1L*, *CC2D2A*, *NPHP3*, *TCTN2*, *B9D1*, *B9D2*, *TMEM231*, *Kif14*, *TMEM107*, *TXNDC15*, etc. [4]. MKS follows the pattern of Mendelian inheritance, whereas non-Mendelian inheritance also contributes to some extent [5]. *CEP290* (OMIM *610,142) locates on 12q21.32(genomic coordinates: 12:88,049,012–88,142,215;GRch38), with a total length of 92.3 kb and contains 55 exons [6]. Mutations of CEP290 lead to a MKS type 4 (OMIM *611,134). *CC2D2A* (OMIM *612,013) locates on 4p15.32(genomic coordinates: 4:15,468,659–15,601,556;GRCH38), 131.5 kb, 38 exons on total, encoding 1620 amino acids [7]. Mutations of *CC2D2A* leads to MKS6 (OMIM *612,284). Furthermore, no clear genotype/phenotype correlations have been clarified nowadays. Some certain inheritance patterns such as basal exon skipping [8], modifier alleles, and stochastic effects might explain this genetic pleiotropy [5], whereas the mutational spectrum still needs to be expanded. From the perspective of histology, MKS is one of the most severe ciliopathies. Primary cilia (PC), a kind of non-motile cilia, play an essential part during fetal growth. Multiple cell signals situate on PCs; one of them is called SHH (sonic hedgehog) pathway; its function consists tightly with cilia [9]. In mammals, SHH is expressed in early embryogenesis and works as a morphogen to pattern the architecture of CNS[10]. In-depth exploration of the relationship between gene mutations and signals will help to clarify the pathogenesis of MKS and is greatly benefit for disease prevention, diagnosis, and treatment.

*ZNF77* maps to the 19th chromosome who carries many ZNF loci and other genes with zinc finger encoding motifs [11]. Zinc finger proteins, combining with some DNAs or RNAs, serves as a transcript factor [12]. Bioinformatic analysis via human tissue-specific networks suggests that ZNF77 controls defensins, elastase, and calmodulin expression, which is firstly described by a research of pathogenic lung colonization [13]. Thus, we suppose that frameshift mutation of *ZNF77* might influence the amount of CEP290 and CC2D2A downstream, leading to alternation of cilia and SHH signal which locate on its membrane.

In this study, we investigated the genealogy of two MKS families. Whole exome sequencing was performed, and mutated locus on *CEP290*, *CC2D2A*, and *ZNF77* were analyzed. Three novel variants were identified to cause MKS and would contribute to its genotype spectrum. Thus, genetic counseling, prenatal diagnosis, and PGT could be benefited referring to a more complete database. Meanwhile, to research the role of ZNF77, we built ZNF77 over-expressed and mutated model in HEK-293 T cell. Through results using western blot, we confirmed a causality between ZNF77 and CC2D2A and a possibility of over-expression or repressed degrade of SHH when *ZNF77* germline is mutated.

## Materials and Methods

### Patients and Families

**Fig.1:**
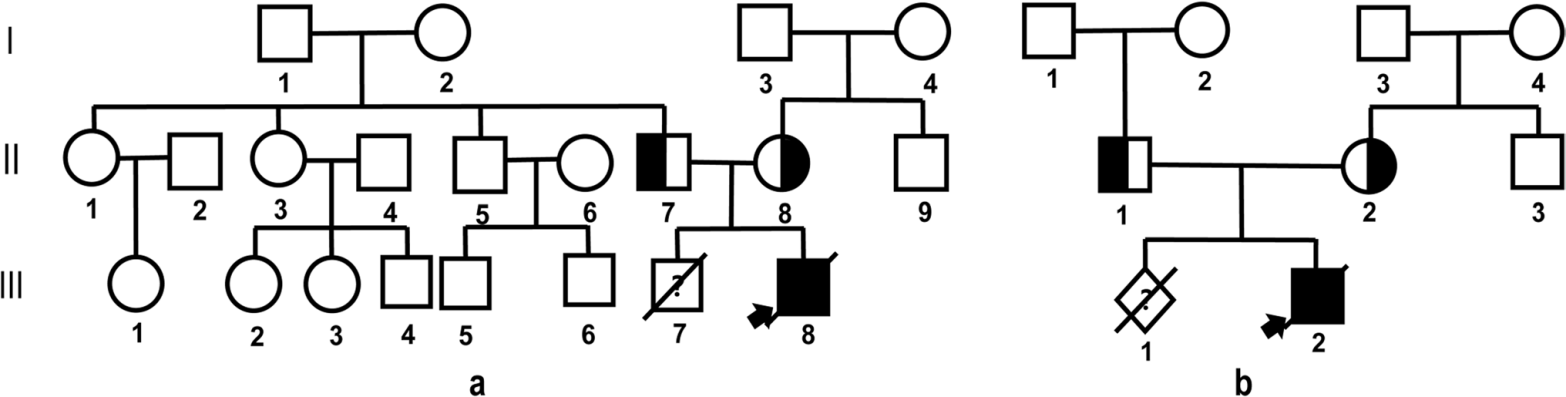
Familial pedigree charts. The arrow indicates the proband, circles indicates female members, square represents males, the slash denotes deceased family member. (**a**) Case 1, II-7, 24 years old, healthy. II-8, 22 years old, healthy. III-7 was induced labor due to MKS; III-8, the proband, showed MKS symptoms (including occipital encephalocele, cystic kidney). (**b**) Case 2, II-1, 28, and II-2, 25; III-1, an aborted fetus of unknown gender, induced labor due to MKS; III-2, the proband, he showed MKS symptoms (occipital encephalocele, cystic kidney dysplasia, postaxial polydactyly)

We investigated two pedigrees diagnosed as MKS, admitted by the Reproductive Medicine Center of Zhongnan Hospital of Wuhan University, Wuhan, China, in January 2019–March 2019. (Fig. 1) Detailed genetic consulting has been performed, and basic information was collected as follows:

In case 1, the mother was 22 years old, and the father was 26 years old. Their first pregnancy was terminated at 24 weeks of gestation with ultrasound findings of facial deformity, encephalocele, and cystic kidney type I and a diagnosis of MKS. No definite mutations and chromosome abnormality was detected from copy number variants (CNV) test of fetus umbilical cord blood. Samples from aborted fetus could not be found by now.

In case2, the mother was 27 years old, and the father is 29 years old. Their first pregnancy ended at 16 WG because of parietal encephalocele, cystic kidney, and polydactyly (suspected MKS), without genetic testing.

**Fig.2:**
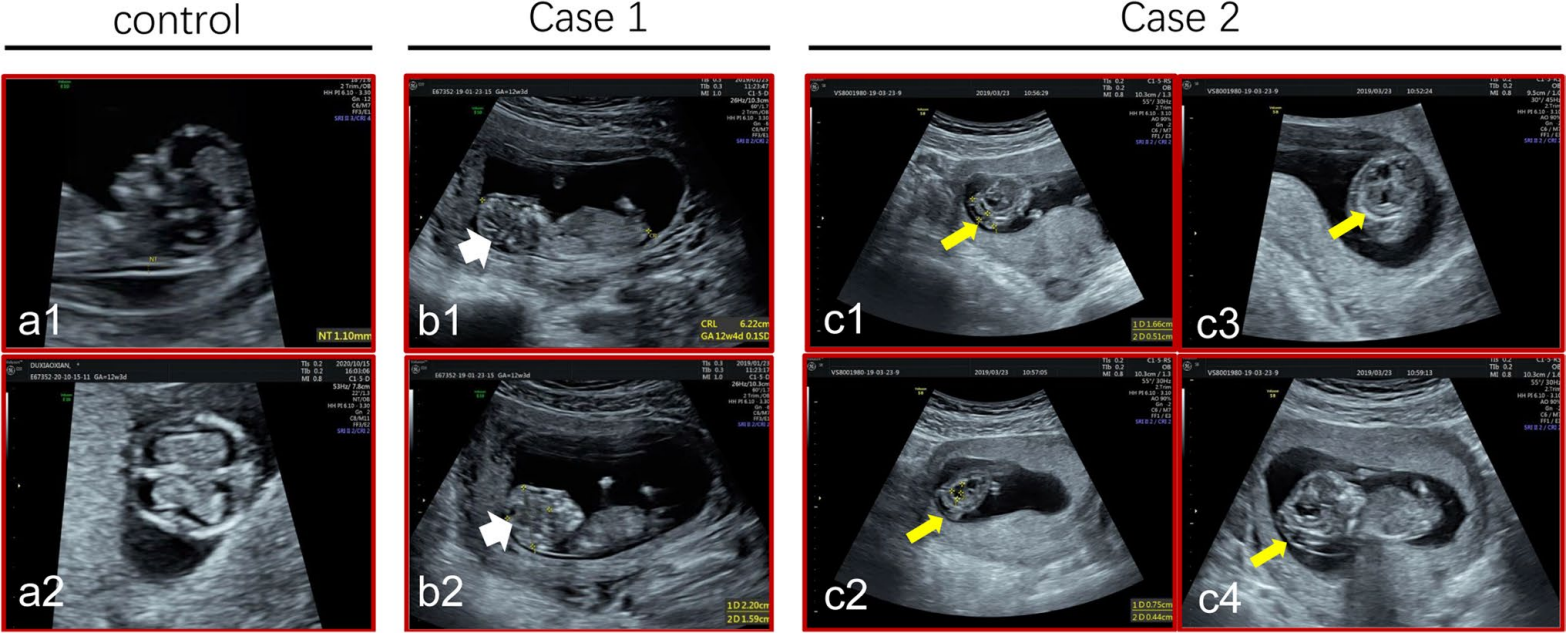
Prenatal ultrasound images. (**a**) Ultrasonography showed the brain of a normal fetus in sagittal section (a1) and in coronal section (a2). The fetus is of 12 weeks gestation. (**b**) Ultrasonography at 12th + 3 week of gestation of the brain of the fetal proband in case 1, in sagittal section. An apical hypoechoic bulge about 2.2 × 1.6 cm covered with entire envelope (occipital encephalocele?), incomplete skull, and mixed intracranial construction can be observed (white arrow in panel b1, b2). (**c**) Ultrasonography at 12th week of gestation of the fetal proband in case 2. An occipital mixed hypoechoic bulge about 1.4 × 0.7 cm in size (occipital encephalocele?) along with incomplete skull can be seen (yellow arrow in panel c1). The midline has disappeared and replaced by multiple anechoic area, one of which is about 0.8 × 0.4 cm

Both two couples were healthy, without markable history of inherited diseases, and denied consanguineous marriage. Encephalocele was found in both families at about 12 WG displayed by ultrasound; the fetuses were aborted at 12 + 5 and 12 + 1 WG, respectively. (Fig. 2) Histological examination, whole exome sequencing, and sanger sequencing were performed.

The research was approved by the Ethics Committee of Zhongnan Hospital of Wuhan University [2020053 K]. We obtained written informed consent from all the participant of this study.

### Methods

#### Whole Exome Sequencing and Bioinformatical Analysis

Two milliliters of peripheral blood was taken from parents; EDTA was used for anticoagulation; 1 g of the hip muscle from the fetus were harvested and minced. Genomic DNA was extracted with the method of phenol and chloroform extraction. High-throughput sequencing platform was used to detect the exon regions and flanking intron regions (20 bp) of 20,999 genes in the human whole exome genome. The results were aligned with the reference sequence of the human genome hg38 (GRcH38). Sanger sequencing were used to verify the mutations of family members according to the screening results (Table 1). Primers were designed by the software Primer5 to amplify the exon regions and splicing regions of mutations in *CEP290*, *CC2D2A*, and *ZNF77* gene. Clinical significance of variants were estimated referring to guideline published by American College of Medical Genetics and Genomics (ACMG).

**Table 1:** Design of primers in sanger sequencing

Gene	Primer	Sequence
CEP290	c2053-F1	aagtcacttgcccagctgtt
	c2053-R1	gcttttgccaactgctgtga
CEP290	c2340-F1	aattcctggcagggcattca
	c2340-seq c2340-R1	ttccagatagaccatcttga tgacaggtgtgcgaacaaga
CC2D2A	CC2D2A-F1	ctctctgctgcctctatgcc
	CC2D2A-R1	gcccctagtggatcataccc
ZNF77	ZNF77-F1	ctctttgctctccaggacgc
	ZNF77-R1	gggatctcactatgcagccc

F1 stands for forward primer, R1 stands for reverse primer. C2340-seq is intermediate primer

The referred database and version of predict software are as follows: ClinVar (2020–03-16), DPS6500 (V2), Thousand Genome (Phase3), GnomAD (r2.0.1), ExAC (r0.3.1), BPGD (V3.1), SecondaryFinding_Var (V1.1_2020.3), dbscSNV (1.1), SpliceAI (1.3), dbNSFP (2.9.1), SIFT, MutationTaste, Polyphen2, Phylop, GERP, etc.

#### Histological Hematoxylin–Eosin Staining Photography

Aborted fetuses were autopsied; their bilateral kidneys, brains, and livers were harvested and stained by hematoxylin–eosin. Tissue blocks were taken to paraffin imbedding and cut into 5 μm sections. The section was placed into xylene to remove the wax, passed through decreasing concentration of alcohol baths (100%, 90%, 80%, 70%) and water, stained in hematoxylin for 3–5 min, and then washed in running tap water until blue. Dip in 1% acid alcohol (1% HCl in 70% alcohol) for a few seconds. Dip in ammonia water until the sections became blue. Stain in 1% Eosin Y for 10 min. The three steps above all follow by tap water washing. Dehydrate in increasing concentration of alcohols. Put slides in two xylene baths for clearing and mount them into DPX. Sections were evaluated with a 200 magnification in the microscope.

#### Western Blot Analysis of Whole-Brain Tissue of the Probands

Whole brains were harvested from each of the fetus, and a normal fetus of 12 WG aborted from an unmarried pregnancy. RIPA lysis buffer containing HALT Protease and phosphatase inhibitor cocktail solution was used to extract proteins. A bead homogenizer was used for homogenization; afterwards tissue lysates were incubated on ice for 1 h. Then centrifuge proteins at 14,000 g for 30 min at 4℃. The supernatant was added with 2 × sample buffer containing 1 mM dithiothreitol. The Pierce BCA Protein Assay Kit was used to estimate protein. Boil samples for 10 min, and take 30 µg to load on 8–12% SDS-PAGE gels. After all protein were transferred onto PVDF membrane, primary antibodies were added and incubated at 4 °C overnight. Then secondary antibodies were incubated at room temperature for 1 h. Membranes are scanned with gel imager (Peiqing equipment, Shanghai), and proteins are visualized and qualified using Image Labe.

#### Construction of Recombinant Plasmid, Transfection, and Western Blot Analysis

First, recombinant plasmid PCDNA3.1 should be generated. DNA primers are designed in accordance with human *ZNF77* gene and Flag sequencing from GenBank database. Restriction enzyme cutting site are added at the both side of primers. Following the instruction, the primers were used as templates for PCR amplification. Products were separated by 1% agarose gel electrophoresis and recovered by a DNA gel recovery kit. Target genes and PCDNA3.1 vector are both digested by restriction enzymes (EcoRI and XhoI) at 37℃ for 2 h. Mix target genes and vector at a ratio of 3:1 and add DNA ligase T4 at a room temperature for 1 h. *E. coli* DH5α are employed to plasmid multiplication. Competent *E. coli* DH5α was transformed with the ligation at 37℃, with 200 r/min and 45 min shaking. The solution of bacteria were cultured at 37℃ in lysogeny broth (LB) medium supplied with 200 μg/ml ampicillin. After overnight culturing, 1% agarose gel electrophoresis and DNA sequencing were performed to verify successfully transformed plasmid.

HEK-293 T cells (ATCC)[14] were cultured in DMEM (11,995,073) supplemented with penicillin (50 μg ml^–1^) and streptomycin (50 μg ml^–1^) along with 10% (vol/vol) FBS and placed into 37℃, CO_2_ 5% incubator. The human embryonic kidney (HEK293T) cell line was obtained from China Center for Type Culture Collection (CCTCC, China, http://cctcc.whu.edu.cn/). The medium was replaced every 2 days. When cell fusion reached about 90%, the cells were digested with 0.25% trypsin, collected, and seeded into 96-well flat-bottom cell culture plates for plasmid treatment at 96 cells/well. Cells were randomly divided into three groups: group with over-expressed *ZNF77*, group with mutated *ZNF77*, and group with empty vector. Transfections were carried out 18–24 h post-seeding with 30 ng PCDNA3.1 plasmid (on the basis of group) and the lipofection reagent (Neofect transfer reagent) 200 μl and buffer 3 μl per transfection and culture for 24 h.

Evaluation and density qualification were performed by western blot as described by previous step.

## Results

### Whole Exome Sequencing and Sanger Sequencing Found Variants in CEP290 and CC2D2A and Frameshift Mutation in ZNF77

**Fig.3:**
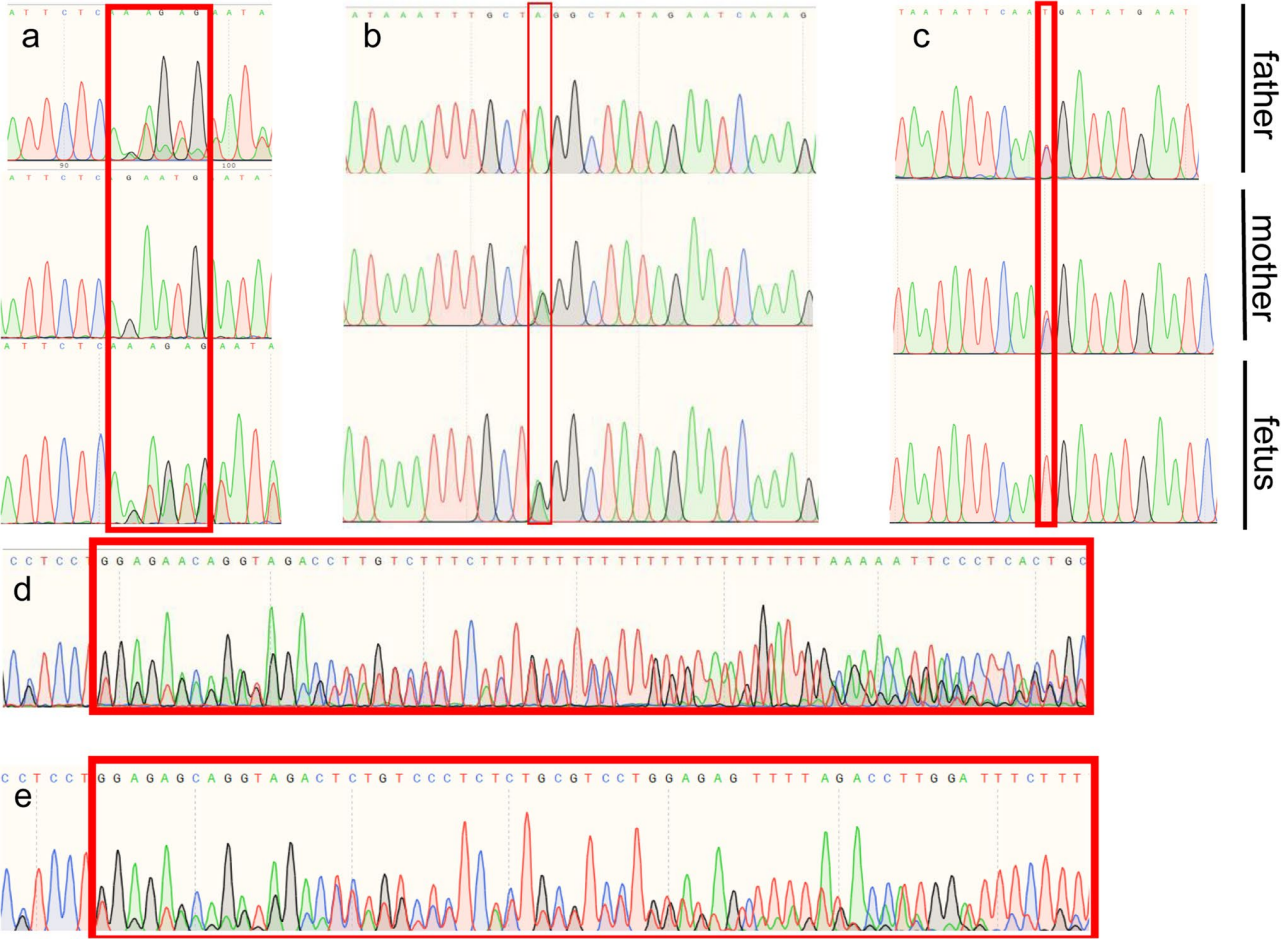
**a**–**c**: Sanger sequencing peak figure. **d**, **e**: Sequence of ZNF77. Variants are noted by red square frame (ps. Frameshift mutation started at chr19:2,939,037. Compared with normal negative chain of chromosome 19.). (**a**) CEP290 c.2340_2341 delGA:p.Asn781*,exon 22. (**b**) CEP290 c.2053-2A > G, IVS20. (**c**) CC2D2A c.4333C > T, exon 35. (**d**) Frameshift mutation of ZNF77 in case 1. (**e**) Frameshift mutation of ZNF77 in case 2

To identify the virulence gene, we performed trio whole exome sequencing and sanger sequencing verification of aborted fetus and their parents. Through WES, the proband of case 1 (III-8) was identified to have a compound heterozygous mutation in *CEP290* gene combining a locus c.2340_2341 delGA (p.Asn781*) and a novel locus c.2053-2A > G. Sanger sequencing confirmed that c.2340_2341 delGA (p.Asn781*) came from the father (II-7); c.2053-2A > G came from the mother (II-8). Sanger sequencing verified their heterozygous state, and they all had a normal phenotype (Fig. 3a, b).

c.2340_2341 delGA(p.Asn781*) locates in exon 22 of *CEP290*, with an estimation as pathologenic (PVS1 + PM2 + PM3_Strong + PP4) on the basis of guideline from The American College of Medical Genetics and Genomics (ACMG)[15]: The variant c.2340_2341delGA (p.Asn781*) located in exon 20 was a frameshift mutation, which is relatively rare. It is predicted that this mutation may lead to the premature amino acid stop code of protein synthesis (PVS1). So far, this mutation has not been reported in the ExAC database and the Thousand People Database (PM2). Morbid variants exist in recessive genetic diseases (PM3). The phenotype and family genetic characteristics of the proband are highly consistent with MKS syndrome (PP4).

c.2053-2A > G is located in the region of intron of *CEP290*. According to ACMG [15], the locus is evaluated as pathogenic (PVS1 + PM2 + PM3): This variant is located in the classical mRNA splicing region, where the nucleotide sequence is highly conserved. It is predicted that abnormal mRNA splicing and editing might happen, and thus, correct mRNA molecule cannot be formed (PVS1). The variant has not been reported in the ExAC database and the Thousand People Database by now (PM2). In recessive genetic diseases, pathogenic variants (PM3) are detected in the transposition.

In addition, we found a novel variant c.4333C > T(p.R1445X)in exon 35 of CC2D2A. Sanger sequencing confirmed the proband (III-2) to be homozygous nonsense mutation and his parents (II-1, II-2) to be in a heterozygous state (Fig. 3c). Likewise, the parents are with normal phenotype. According to ACMG [15], the locus has been considered as pathogenic (PVS2 + PM2): The variant locates on exon 35 and leads to a nonsense mutation and thus a premature stop codon. Nonsense-mediated decay might account for the lethal phenotype. In addition, it is known that certain loss-of-function (LOF) mutation have already been reported (PVS1). The locus has not been reported in any related literature nor was it included in the gnomAD database (PM2).

In addition, frameshift mutations were detected in exon 2 of *ZN77* in both probands (Fig. 3d, e).

### Basic Conditions of Patients, Ultrasound, Gross Specimen, and Pathological Examination

**Fig.4:**
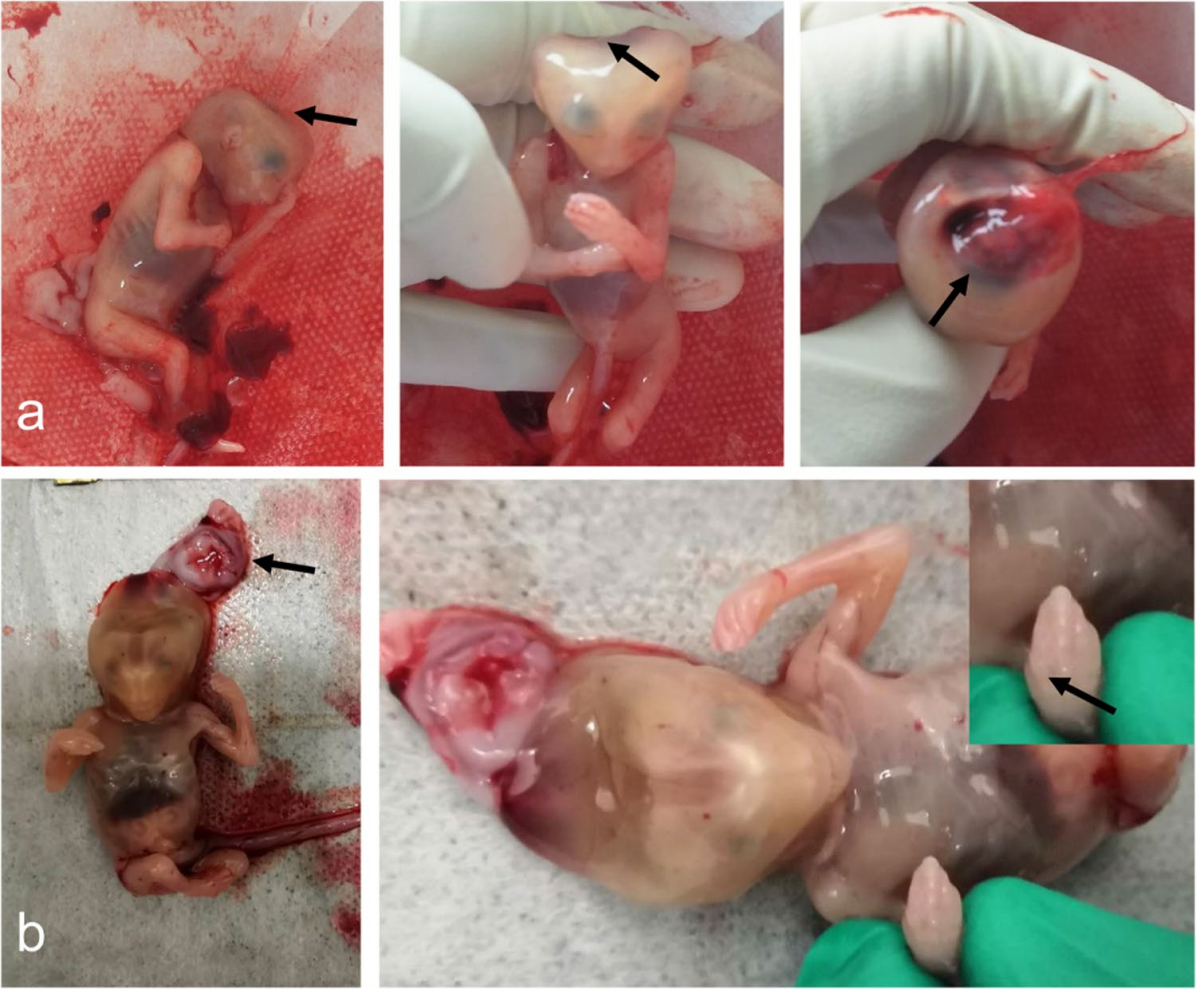
Clinical photographs. (**a**) Proband in case 1, fetus aborted at 12th + 5 week of gestation. The skull is defect, and the brain tissue can be seen (black arrow). (**b**) Proband in case 2, fetus aborted at 12th + 1 week of gestation. The picture showed encephalocele and polydactyly (black arrow)

**Fig.5:**
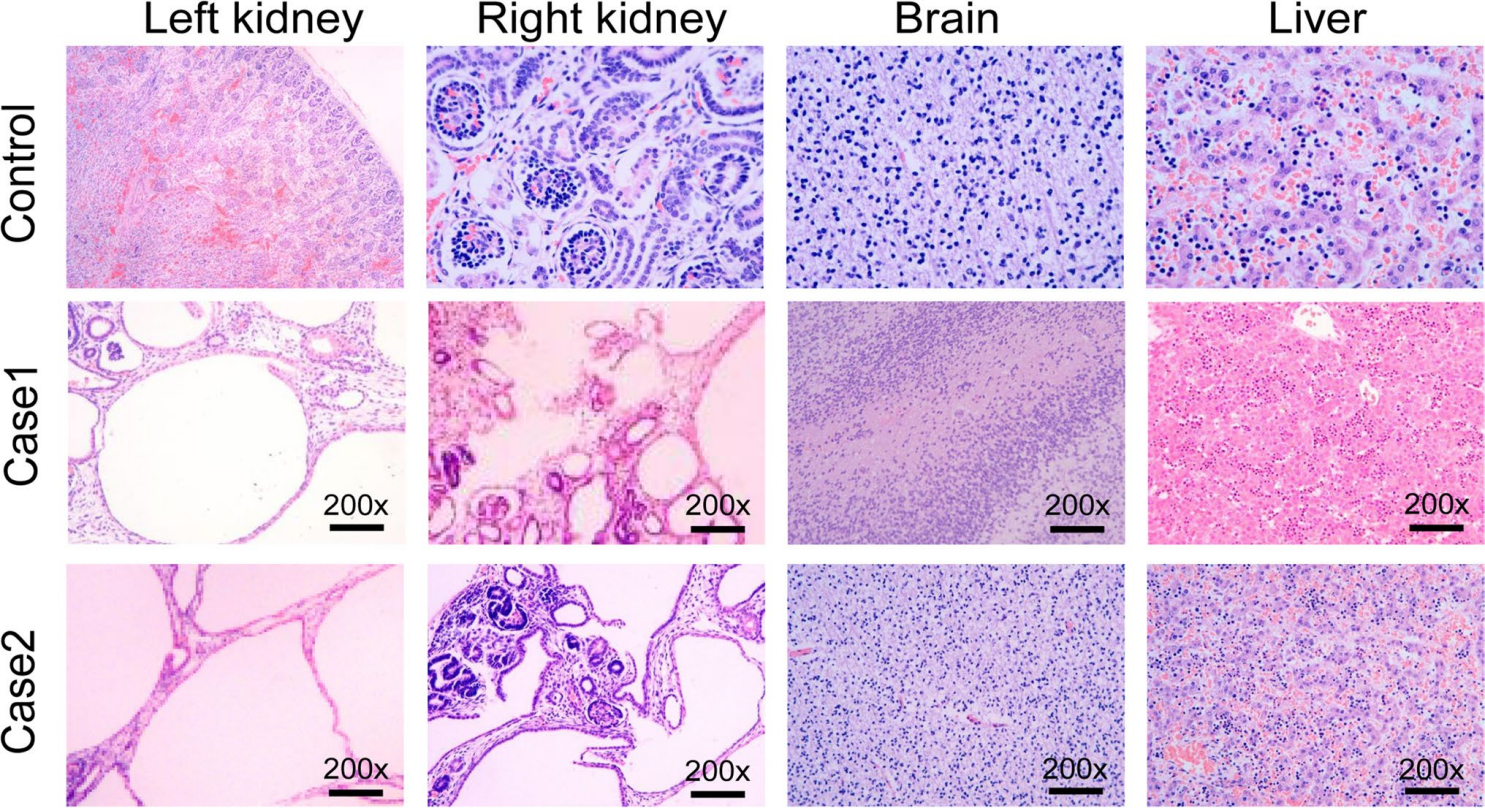
Histopathological examination under microscope of essential organs. Normal tissues have been obtained from fetus of 12 weeks gestation and without relative disease. (**a**) Fetus proband in case 1. Histopathological images showed structure of primary glomerulus and renal tubules in both bilateral kidneys, part of those tubules undergoing cystic dilatation (black arrow in panels 1, 2). Local brain tissue edema and neuronal degeneration within neurons, glial cells, and primitive neural tubes (black arrow in panel 3). Hepatic cells are normal (panel 4) HE staining. (**b**) Fetus proband in case 2. Extensive cystic renal tissues can be observed, without obvious cortex and medulla, on both side of the kidneys (black arrow in panels 1,2). Image of encephalocele tissues could not find stratification (black arrow in panel 3). Hepatic cells are normal (panel 4). HE staining

In case 1, specimen of aborted body showed that the fetus was 9.5 cm in length and 21.1 g in weight. The head circumference was about 7 cm, and a 1.5 × 1 cm defect was located on cranial bone, from which part of the brain tissue weighing 1.3 g bulge out. Other brain tissue weighs 1.3 g which remain in the skull, with a mixture of structure and vaguely recognizable cerebrum and cerebellum (Fig. 4a). Hematoxylin–eosin staining pathological images of the brain demonstrated local edema, and neuronal degeneration within neurons, glial cells, and primitive neural tubes can be seen as well. Those kidneys demonstrated primary glomerulus and renal tubules especially the later undergoing cystic dilatation (Fig. 5).

In case 2, the gross specimen appeared 9 cm in length and 18.7 g in weight; the head circumference was 6.0 cm, biparietal diameter is 2.0 cm. The fetus had facial and skull hypoplasia, and part of the brain bulged from the cranial notch, about 3.0 × 2.0 × 0.3 cm in volume and 7.0 g in weight. The size of left kidney is about 2.0 × 1.0 × 1.8 cm, and the right is 2.0 × 1.0 × 1.8 cm; altogether the kidneys weigh 1.8 g; extra fingers and toes could be seen on his limbs (Fig. 4b). HE staining pathological images demonstrated concentrated neurons and inapparent stratification; cystic dilatations without obvious cortex and medulla, adrenal could not be found (Fig. 5).

### Expression CEP290, CC2D2A, and ZNF77 Were Enhanced in MKS Fetus

**Fig.6:**
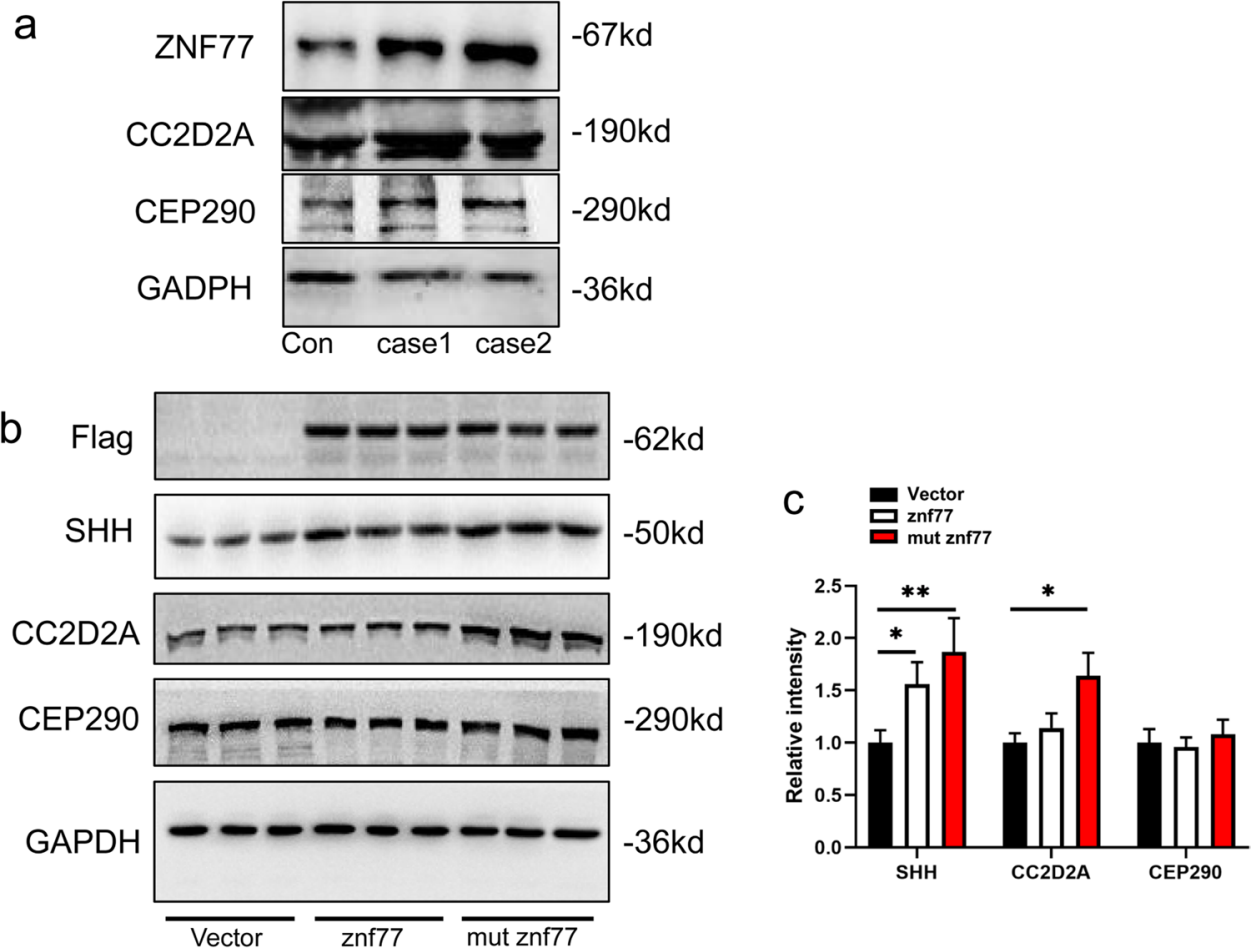
Western blotting. Numbers on the right indicate molecular weight. (**a**) Western blot analysis of whole-brain tissue from a normal fetus and probands in case 1 and case 2. Compared with control, the density of ZNF77.CEP290 and CC2D2A appeared to decreased. (**b**, **c**) Western blot analysis of Flag-tagged ZNF77 and Flag-tagged mutated *ZNF77* transfected HEK293cell cell. Lanes 1–3 demonstrate empty plasmid as control, with lanes 4–6 demonstrating *ZNF77*-transfected cells and lanes 7–9 mutated *ZNF77*-transfected. Density of the proteins is directly shown by relative intensity in histogram. Remarkable increase is noted by asterisk above cross line; the more asterisks the stronger increase. SHH has increased when *ZNF77* was transfected and enhanced more significantly when mutated *ZNF77*. CC2D2A showed notable increased when mutated *ZNF77* was transfected. CEP290 has no obvious change

We performed western blot utilizing whole-brain tissue from two fetus. In comparison with normal brain, both patients (lane 3 and lane 4) had higher density of CC2D2A an ZNF77, suggesting that the expressions of CC2D2A and ZNF77 were increased (Fig. 6a).

### ZNF77 Plasmid Transfected HEK293T Cells to Convince Correlations Between ZNF77 and CC2D2A, SHH

A histogram was used to quantify the density; remarkable increasing was noted by asterisk above cross line; the more asterisks the stronger increased. Compared to cells transfected by empty vector, SHH had increased in ZNF77 over-expressed cells; nevertheless, CC2D2A and CEP290 did not alter significantly; in *ZNF77* mutated cells, SHH had a higher enhancement, along with a notable enhancement of CC2D2A and stable CEP290 (Fig. 6b, c). In other words, over-expressed ZNF77 might promote the expression of SHH; mutated ZNF77 might enhance SHH as well as CC2D2A.

## Discussion

MKS is categorized as ciliopathies and represents the most serious conditions. Ciliopathies present a general term of diverse monogenetic disorders displaying abnormal developmental and degenerative symptoms, caused by a hair-like cellular organelle, cilium [16]. German physicist Johann Friedrich Meckel had firstly reported the disease in 1882; he described two siblings with typical features: occipital encephalocele, cystic kidneys, and polydactyly [17]. Later an investigator Gruber noted the same condition [18]. MKS has been reported more frequently since 1960s, and then, gradually up-to-date diagnostic criteria has been refined and abstracted. We referred to a review [19] to make a diagnosis of MKS. Nowadays we acknowledge a classical triad (occipital encephalocele, polydactyly, cystic kidney) as recognizable character of MKS [19], yet it is often controversial whether a diagnosis of MKS should be provided with all of the followings. After all, penetrance rates are not consistent in different case reports, classical triad do not always exist in one case. In our case 1, polydactyly does not appear. Thus, iconography is considered not to be the unique method to detect a MKS fetus; the importance of molecular DNA testing has been emphasized. Meanwhile, since MKS is a rare inherited disease, we could not get plenty of samples. Correspondingly, every sample is important for the research of the disease.

In cilium, a region at the base that links axoneme microtubules and surrounding membranes is called “transition zone” (TZ). Multiple proteins situated in TZ and formed functional complexes, consisting MKS module and NPHP module [20]. Centrosomal protein of 290 kDa (CEP290) locates in TZ, centrosome/basal bodies, and pericentriolar satellites [21, 22]; interacts with other components in TZ; and together serves as barriers at ciliary gate [23].

Mutations of CEP290 lead to a continual phenotypic spectrum, from Leber congenital amaurosis (LCA) which is least severe, Joubert syndrome (JBTS) which is middle severe, to MKS type4, the most severe. Few cases have been reported about CEP290-related MKS. The first variation c.2144 T > G(p.Leu715*) homozygous mutation that causes MKS4 in Chinese population is identified in 2019 [24]. In their case, the proband displayed occipital encephalocele and cystic dilatation, thus diagnosed as MKS and aborted.

**Fig.7:**
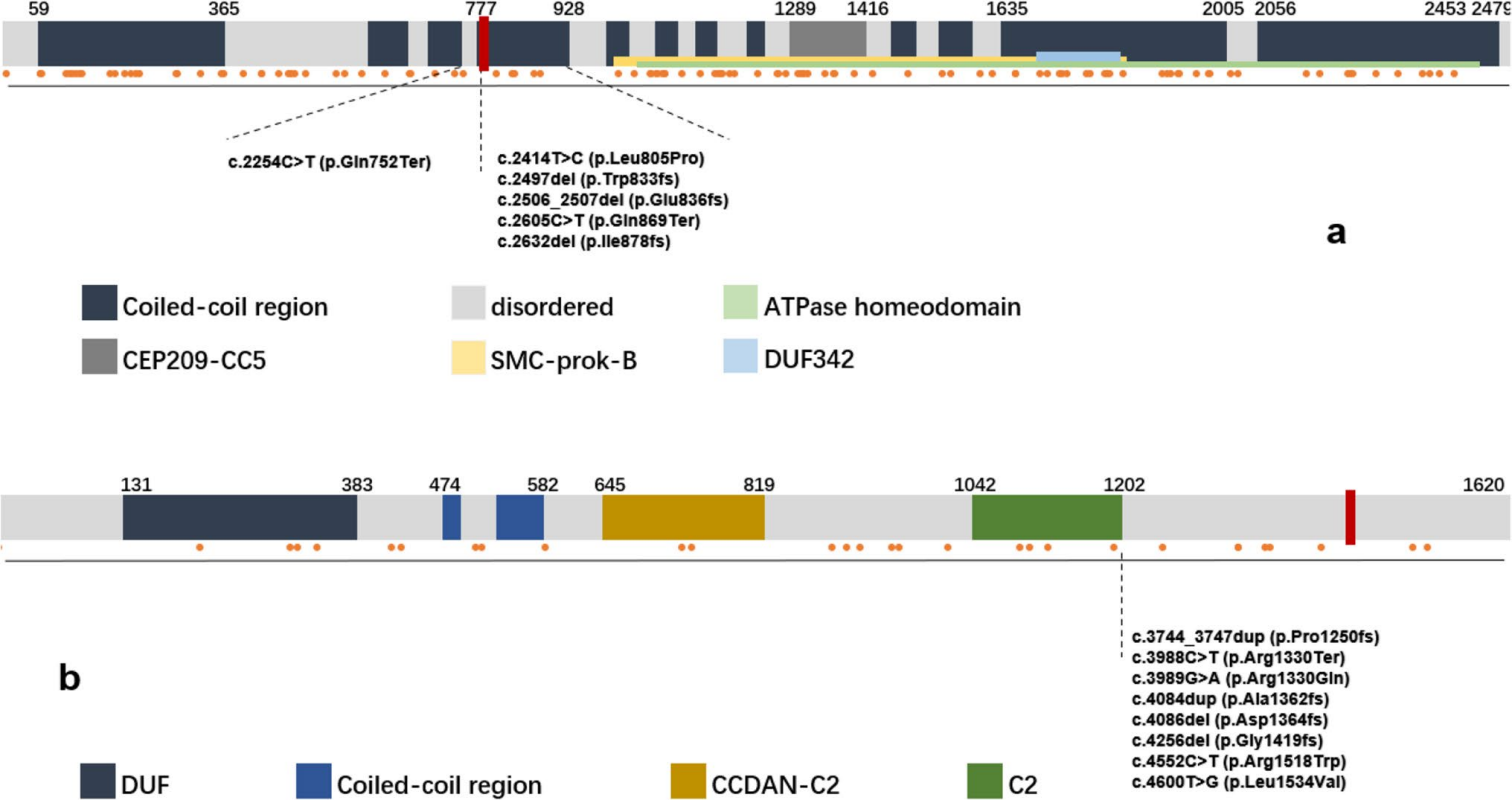
A map of reported pathogenic variants of MKS. Domains are shown by the strip above and exons are shown below. Orange spots in the middle stand for variants we have counted. We denoted our novel variants by red square box at the position corresponding its location in real amino sequence. (One of them is in intron region so it hasn’t been marked.) Surrounding variants are written out to prove that our variants are novel. (**A**) CEP290. (**B**) CC2D2A. Variant information were downloaded from NCBI ClinVar database, and domain information were referred to NCBI protein database, Pfam database, and SMART database

In our case 1, the variant c.2340_2341 delGA (p.Asn781*) is a frameshift mutation. Yan Xu et al. had reported correspondence with LCA in 2016 [25]. Lushan LI et al. had reported in two compound heterozygous fetus. Both cases contained the variant; the probands had manifestations of enlarged ventricles, bilateral polycystic kidney dysplasia, and oligohydramnios. They did not show encephalocele; thus, they had not been diagnosed as MKS [26]. In brief, in our case, this variant firstly appeared in a MKS patient. (Fig. 7a).

Coiled-coil and C2 domain-containing 2A (CC2D2A) is a member of MKS/B9 module. It contains 3 possible coil domains, a C-terminal C2 domain, a potential CaMKII recognition site, and a PKC phosphorylation site, and 2 possible nuclear localization signals [27]. CC2D2A is a component of the basal body protein complex, forming a ring structure that functions in the transition zone at the base of the cilia. This complex serves as a barrier that restricts the diffusion of proteins between the plasma and the ciliary membrane [28]. In our case 2, the novel variant c.4333C > T (p.R1445X) was located on the 35 exon. A premature stop codon that initiated nonsense-mediated decay might account for the lethal phenotype [29]. The variant we have found in CC2D2A is novel as well as the variants in CEP290 (Fig. 7b).

Western blot analysis of the brain tissue from both probands showed enhanced CEP290, CC2D2A, and ZNF77 in case 1 and case 2 (lane 2 and lane 3). However, as a 100% mortality rare inherited disease, with an early gestational age, we could not get more tissues. Thus, we could not perform statistical analysis.

In addition to three novel variants, our study brings up an effect of ZNF77 in certain MKS-related gene expression and signal pathway. *ZNF77* maps to 19 chromosome which carries many ZNF loci and other genes with zinc finger encoding motifs [11]. With a role of transcript factor, proteins of zinc-finger families could combine with DNAs and RNAs [12]. Bioinformatic analysis via human tissue-specific networks suggests ZNF77 controls defensins, elastase, and calmodulin expression, which is firstly described by a research of pathogenic lung colonization [13]. Other literatures brought forward its contributions to early-onset dyslipidemia, myocardial infarction and chronic kidney diseases, etc. [30]. Our research firstly discovered mutations in ZNF77 might have effects on embryogenesis. HEK-293 T cell serves as a widely used cell model; SHH plays a role of a signal molecule from the start of SHH pathway. Its enhanced expression might represent an activated pathway. To further investigate the correlation between ZNF77 and proteins above, we combined ZNF77 and mutated ZNF77 with PCDNA3.1 plasmid to build over-expressed ZNF77-PCDNA3.1 plasmid and mutated ZNF77-PCDNA3.1 plasmid. Afterwards we transfected HEK293T cells with those two plasmids and empty vector to construct over-expressed ZNF77 model and mutated ZNF77 model. Then we proceeded western blot of lysates of HEK293T cells to estimate altered expression of target proteins. Targeted proteins are tagged by Flag; a histogram is used to quantify the density; remarkable increase is noted by asterisk above cross line; the more asterisks the stronger increase. Western blot of transfected HEK293T cells revealed that mutated ZNF77 instead of normal or over-expressed ZNF77 could enhance expression of CC2D2A. We suppose that active ZNF77 regulates the amount of CC2D2A to remain stable, possibly inhibits it from over-expressing. Unaltered CC2D2A in ZNF77-over-expressed cells reveals that the inhibition has nothing to do with normal amount of active ZNF77, as well as an excess. CEP290 staying changeless interprets no markable correlations thereof.

In mammals, Hedgehog is classified into 3 types, desert hedgehog (DHH), Indian hedgehog (IHH), and sonic hedgehog (SHH) [31]. During neurogenesis, SHH plays a role of morphogen, mitogen, and guidance molecule [32]. Lacking of SHH might result in neural tube dorsalization and polydactyly [9]. From embryo period to postnatal growth, SHH constantly takes effects in cerebellar pattering and maturation. Its abnormal expression not only leads to embryo neural defects but contributes to serious neural diseases, such as Parkinson’s disease, autism spectrum disorder, depression, dementia, and stroke as Sita et al. has reviewed [33]. SHH works as the start of SHH signal, primarily being translated into 45 kDa precursor who shortly afterwards auto-proteolytically cleaved into signaling active N-terminus and inactive C-terminus [34]. SHH diffuses from localized source to distant cells to elicit transcriptional responds through ligand that lying on ciliary membrane [35]. Some certain cancers including medulloblastoma (MB) [36] and carcinoma basal cell [37] are mediated by overactive SHH pathway; thus, in neurons, activation of SHH might induce neural recovery. Shh plays a role in the proliferation of cerebellar granule neuron precursor (CGNPs) and regulates its cell cycle by upregulating or maintaining cyclin gene and cyclin-Rb axis in G1 [38]. SHH binds to its specific receptor Ptch1 [39] on ciliary membrane and co-receptors Cdo/Boc and then undergoes endocytosis and lysosomal degradation mediated by Megalin to negatively feedback its concentration [40]. In our study, over-expression of ZNF77 caused a corresponding increase in SHH expression and inactivated ZNF77 protein even greater. We suggest that the enhancement might attribute to a promoted expression or an inhibited degradation, probably not CC2D2A. To be sure, ZNF77 is a factor who effects on SHH.

Taken together, mutated ZNF77 can enhance the expression of CC2D2A as well as SHH, whereas over-expressed ZNF77 simply increases that of SHH. We could come up with a hypothesis of causality between ZNF77 and CC2D2A/SHH, especially a role of impacting factor in SHH pathway. Yet there is other approaches excluding CC2D2A for ZNF77 to effect on SHH pathway. Glioblastoma (Gli) proteins are downstream effectors which contains zinc-finger domains[41], aligning with the same function of ZNF77. More in-depth researches of gene expression context and whether there are some feedback mechanisms can be done in the future.

In conclusion, MKS is a severe inheritance disease which cannot be ignored due to its low incidence. Recently reports had gradually increased along with the development of molecular genetics. Our research also brings about several reasons serving as evidence for prenatal diagnosis of MKS. By now, single gene testing has low sensitivity because of the absence of clear genotype–phenotype correlations for MKS. However, once a morbid variant in a family has been identified, prenatal genetic diagnosis by placental chorionic sampling can be prioritized during subsequent pregnancies. PGT might also be used to optimize embryos, then decrease the risk of induction of labor, and elevate the health quality of population. In addition, the discovery of new mutation sites has enriched the MKS pathogenic genotype spectrum. We have also found the potential effect of ZNF77 on CC2D2A and SHH pathways. All of above have laid a good foundation for the follow-up research on the pathogenic mechanism of MKS.

## Data Availability

All data generated or analyzed during this study are included in this published article.
